# Congenital Osseous Torticollis that Mimics Congenital Muscular Torticollis: A Retrospective Observational Study

**DOI:** 10.3390/children7110227

**Published:** 2020-11-13

**Authors:** Da-Hye Ryoo, Dae-Hyun Jang, Da-Ye Kim, Jaewon Kim, Dong-Woo Lee, Ji-Hye Kang

**Affiliations:** Department of Rehabilitation Medicine, Incheon St. Mary’s Hospital, College of Medicine, The Catholic University of Korea, Seoul 06591, Korea; neatlisa05@gmail.com (D.-H.R.); mnikdy@gmail.com (D.-Y.K.); jw2356@naver.com (J.K.); violetfear1@naver.com (D.-W.L.); rkdwlgp91@naver.com (J.-H.K.)

**Keywords:** congenital torticollis, cervical vertebrae, congenital abnormalities

## Abstract

It may be difficult to diagnose congenital osseous torticollis based on physical examinations or plain X-rays, especially when children have no other accompanying congenital defects. This study reports the children with torticollis caused by the vertebral anomaly with the symptom of abnormal head and neck posture only. We retrospectively reviewed the records of 1015 patients diagnosed with congenital torticollis in a single tertiary hospital (Incheon St. Mary’s Hospital, Korea) who were referred from a primary local clinic. We included those with deficits in passive range of motion (PROM) of neck. Ultrasonography of the sternocleidomastoid (SCM) muscles, ophthalmologic and neurologic examinations, and cervical X-rays were performed for all patients. If bony malalignment was suspected from X-ray, three-dimensional volume-rendered computed tomography (3D-CT) was performed. Ten patients were diagnosed with osseous torticollis with no defect other than bony anomalies. Although X-ray images were acquired for all patients, vertebral anomalies were definitely confirmed in three cases (30.0%) only, and the others (70.0%) were confirmed by CT. The most common type of vertebral anomaly was single-level fusion. Identifying congenital vertebral anomalies is challenging especially when the degree of invasion is only one level. Although abnormal findings on X-rays may be subtle, a careful examination must be performed to avoid misdiagnosis.

## 1. Introduction

Normal head and neck posture is defined as having the mouth horizontal and the nose vertical with respect to the horizontal plane [1,2]. The abnormal posture of the head and neck refers to torticollis and is often encountered in the pediatric clinic. Congenital muscular torticollis (CMT) is the most common cause of torticollis in children [3]. Therefore, although congenital torticollis can be caused by congenital structural abnormalities of the vertebrae, it can be misdiagnosed as CMT on the first clinic visit. Moreover, congenital vertebral malformation may be difficult to diagnose based on physical examinations or plain X-rays. For these reasons, osseous torticollis is likely to be overlooked especially if vertebral anomalies are not accompanied by other discernible congenital defects, such as vertebral defects, imperforate anus, tracheoesophageal fistula, and radial and renal dysplasia (VATER) syndrome; Klippel–Feil syndrome (vertebral fusion with prominent features such as low hairline, short neck, facial asymmetry, scoliosis, or spina bifida and genitourinary problems, heart abnormalities, or lung defects); Alagille syndrome; or Müllerian duct aplasia, renal aplasia/agenesis, and cervicothoracic somite dysplasia (MURCS) association. Vertebral anomalies increase instability risk and spinal cord encroachment and therefore should be considered in pediatric patients with abnormal head and neck posture to avoid potential neurologic injury [4,5,6,7,8]. If a correct diagnosis is confirmed, patients can receive proper management and clinicians can avoid pursuing unnecessary physical therapy. However, the lack of information dealing with spinal malformations that only present as an abnormal head and neck posture leads clinicians to misdiagnose it as the most common diagnosis, CMT, rather than osseous torticollis. In this study, we report the children with torticollis due to asymptomatic vertebral anomalies except for abnormal head and neck posture. Thus, we emphasize that there should not be cases where unnecessary active physical therapy is done to the osseous torticollis and, in particular, surgery such as muscle relaxation is done to the osseous torticollis with misdiagnosis as CMT that does not respond to physical therapy.

## 2. Materials and Methods

We retrospectively analyzed the medical records of patients diagnosed with congenital torticollis at a single tertiary hospital from 2011 to 2019. The inclusion criteria were limitation in the passive range of motion (PROM) of neck rotation and/or tilting with suspicion of congenital torticollis among children who were referred to the tertiary hospital from a primary local clinic. Exclusion criteria included no limitation of neck ROM or the presence of fracture, dislocation, or subluxation of the spine. In this study, children with acute torticollis including atlantoaxial rotatory subluxation, such as Grisel syndrome, were excluded because the diagnosis is not congenital.

This study was approved by the institutional research review board of the Catholic University of Korea, Incheon St. Mary’s Hospital (number: OC19RESI0143). The requirement for informed consent from individual participants was waived because the study was retrospective and did not manipulate the environment or subjects, and because relevant data were obtained from medical records. 

Patients with suspected congenital torticollis were referred to the torticollis center of our hospital from a primary local clinic. Patients who met inclusion criteria based on physical examination, as our clinical pathway of torticollis evaluation, underwent an ultrasonographic (USG) examination of the sternocleidomastoid (SCM) muscle and received ophthalmologic screening and neurologic examinations by one experienced pediatric physiatrist, who was specialized in torticollis. Cervical X-ray images (anteroposterior and lateral view) were also obtained. In patients in whom there was a suspicion of visual abnormalities such as ocular misalignment, nystagmus, or strabismus, the physiatrist requested a consultation to the ophthalmologist for further evaluation. If normal cervical alignment and morphology were not evident on X-rays, and USG did not show any abnormality, three-dimensional volume-rendered computed tomography (3D-CT) of the cervical spine was performed under suspicion of vertebral anomaly [9]. 

From the above process, patients with congenital torticollis were divided into the following four groups: ocular torticollis, defined as torticollis caused by ophthalmic conditions, such as disorders of ocular movement and nystagmus [10,11]; neurogenic torticollis, defined as abnormal head and neck posture due to associated neurologic abnormalities that weakens the neural connections required for proper head and neck positioning (e.g., cerebral palsy) [11,12]; osseous torticollis, defined as torticollis associated with congenital malformation of the spine such as vertebral segmentation defects [11,13,14,15]; and CMT, diagnosed by evidence of shortening of the SCM muscle via physical examination and/or enlarged thickness, heterogeneous echogenicity, fibromatous lesion, or asymmetry of the SCM muscle on sonographic findings. The diagnosis of CMT included tumor, muscular, and postural types [16,17,18,19,20,21,22,23]. 

Patients initially diagnosed with CMT received physical therapy. Manual stretching, strengthening exercises using postural reactions or visuo-auditory stimulations by physiotherapists, and the patient’s family were included in the physical therapy. A well-trained, experienced physiotherapist administered a standardized protocol for manual stretching and strengthening exercises 2 or 3 times weekly. All children were followed up at our hospital at 1-month intervals, until the final assessment. The therapy was discontinued when patients achieved full PROM of the neck and showed no tilting clinically. Patients initially diagnosed with CMT but who did not respond to physical therapy were reevaluated to determine whether other causes of abnormal head and neck posture were present, by the same doctor who made the first evaluation. 

## 3. Results

**Figure 1:**
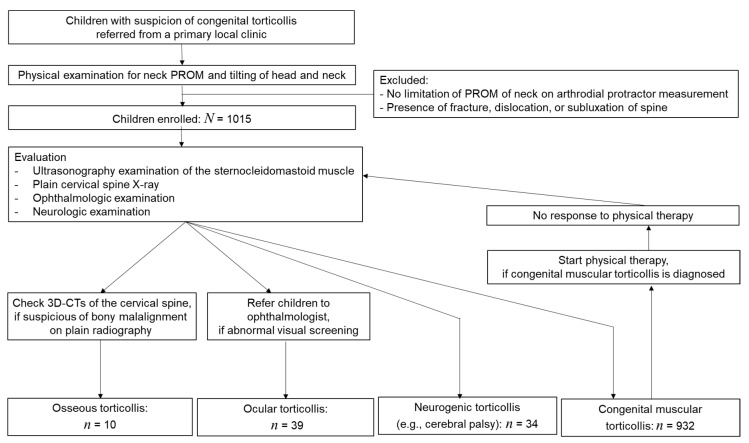
Flowchart detailing patient enrollment. Abbreviations: 3D-CT, three-dimensional volume-rendered computed tomography; PROM, passive range of motion.

Of 1015 patients, 932 (91.8%) were diagnosed with CMT, 34 (3.4%) were categorized into the neurogenic torticollis group, 39 (3.8%) into the ocular torticollis group, and 10 (1.0%) patients into the osseous torticollis group (see Figure 1).

**Figure 2:**
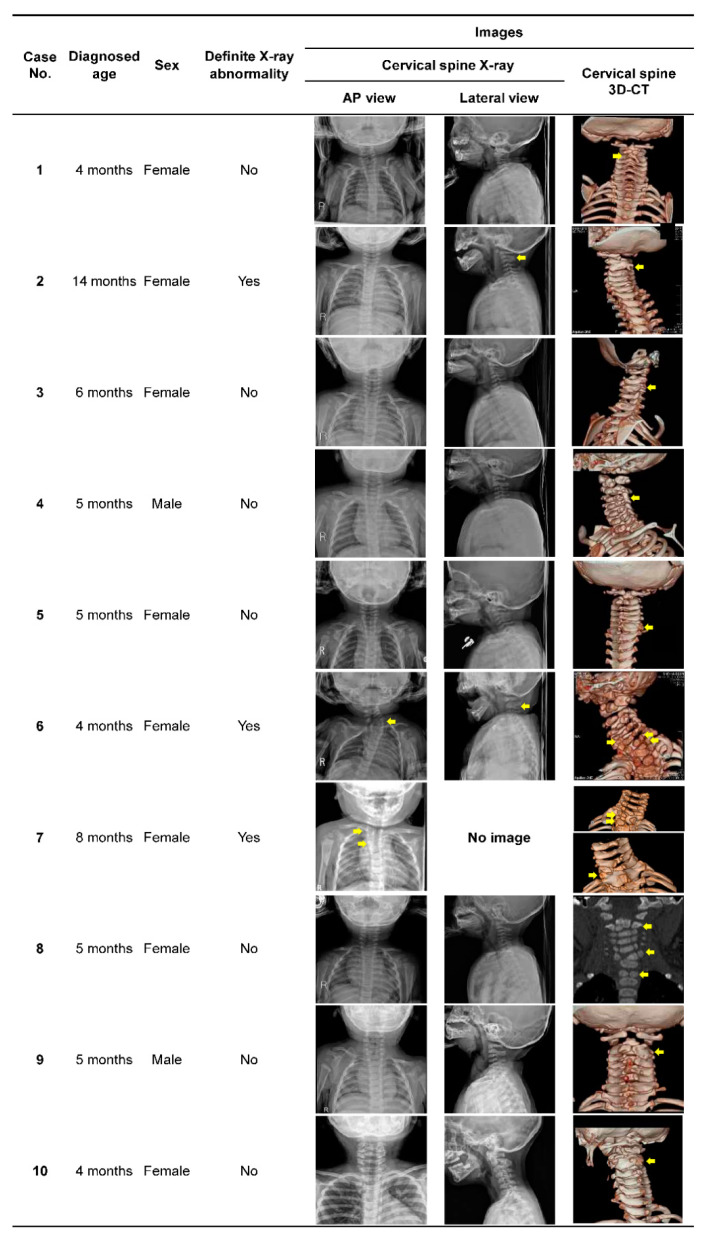
Summary of patients’ characteristics and radiologic features of cervical spine anteroposterior and lateral X-ray and 3D-CT (arrows indicate vertebral anomaly). Representative images of 1. C2‒C3 vertebra fusion; 2. C2‒C3 vertebra fusion; 3. C3‒C4 vertebra fusion; 4. C3‒C4 vertebra fusion; 5. C6‒C7 vertebra fusion; 6. C5 and T1 hemivertebra, C7 butterfly vertebra; 7. T1 hemivertebra, T2 butterfly vertebra, T1‒T2 vertebra fusion; 8. C2 supernumerary hemivertebra, C6 and T1 butterfly vertebra; 9. C2‒C3 vertebra fusion; 10. C2‒C3 vertebra fusion. Abbreviations: 3D-CT, three-dimensional volume-rendered computed tomography.

The average age of the 10 patients in the osseous torticollis group was 6.0 ± 3.1 months (range, 1–14 months). Nine patients (90.0%) were diagnosed before one year of age, and the male-to-female ratio was 1:4 (see Table 1). In the examination for other combined congenital defects, no problem other than vertebral anomaly was detected. Although plain X-ray images were acquired for all patients, vertebral anomalies were definitely confirmed by X-ray in cases 2, 6, and 7 (30.0 %), and the remaining seven (70.0%) were confirmed by CT (see Figure 2). Among the 10 patients, seven (70.0%) had vertebral fusion only without other accompanying deformities. Three patients (30.0%) showed multiple vertebral anomalies in the combination of vertebra fusion, hemivertebra, or butterfly vertebra. The most common type of vertebral anomaly was single-level fusion, and the most frequently involved levels were C2 and/or C3. Of the 10 patients, seven patients (70.0%) had only one level of vertebral involvement, and seven (70.0%) had involvement of the C2 and/or C3 vertebrae (see Table 2; Figure 2).

In case 8, the child was initially diagnosed as CMT, but later diagnosed as osseous torticollis. Upon initial evaluation, no definite abnormality was detected on cervical X-ray and ophthalmologic evaluation, hence physical therapy was initiated with the diagnosis of CMT (see Figure 2). However, no improvement was observed after one month of treatment. About that time, while taking a bath, the child’s neck became overextended. Neck CT was performed in the emergency department of another hospital, which suggested hemivertebra. We reevaluated the patient and performed magnetic resonance imaging (MRI) of the cervical spine to assess the spinal cord injury and vertebral anomaly. Although there was no spinal cord injury, the MRI data revealed the C2 supernumerary hemivertebra and the butterfly vertebra of C6 and T1.

## 4. Discussion

Abnormal head and neck posture could be the only detectable symptom of a congenital vertebral anomaly. Because CMT is the most common cause of torticollis in children and is evident at birth or shortly thereafter, the vertebral anomaly is often overlooked as a cause of torticollis [20].

In the present study, we evaluated children with congenital torticollis who were referred to our hospital from a primary local clinic because of abnormal head and neck posture and found that 10 of the 1015 (1.0%) cases were due to congenital structural deformities of the vertebra. Our results reflect those of several previous studies, which raise clinicians’ awareness of vertebral anomaly as a cause of torticollis. In particular, the studies support the fact that, although patients have vertebral anomalies, the only symptom can be torticollis [3,4,24,25,26]. If patients with congenital vertebral anomaly have no symptoms that must be resolved for daily life, such as pain, they do not need overtreatment [27,28]. Instead, they should be closely monitored for indications of surgery such as severe mechanical neck pain, neurologic compromise, and progressive deformities because congenital malformation can progress (i.e., cervical hemivertebra with progression at 1° to 3.5° per year) and the progression can induce such symptoms [27,28,29,30]. In addition to altered spine kinematics due to cervical deformity, the children have specific biomechanics of the vertebral column due to a relatively heavy head, weaker neck muscles, and vertebral ligament elasticity. In this situation, the risk of progression can increase with external force [31,32]. Thus, our patients who were diagnosed as congenital osseous torticollis did not receive active physical therapy. None of the patients had neurologic signs, and there was no suspicion of spinal cord injury. Therefore, they did not undergo surgery at the time of diagnosis. From the first diagnosis, we have followed up these patients regularly to determine if there is an aggravation of the deformity or the presence of complications such as spinal cord encroachment. Thus far, no patient has shown any additional complications, and none have required surgical intervention.

The osseous torticollis group of the present study showed no other specific features except for abnormal head and neck posture upon first referral to our hospital from a primary local clinic. Therefore, our center’s evaluation protocol included USG, cervical X-ray, ophthalmologic examination, and, if necessary, 3D-CTs, which were needed for diagnosis. Moreover, if patients who were initially diagnosed as CMT but showed no response to physical therapy, the vertebral or ophthalmologic re-evaluation was important (see Figure 1). Such as in case 8, when clinicians evaluate children with torticollis, and although abnormal findings may be subtle on cervical X-rays, they should consider the possibility of bony malalignment and perform a careful examination so as not to miss the diagnosis.

The frequency of congenital torticollis due to the vertebral anomaly is not clear. According to the studies by Tomczak et al. and Haque et al., non-muscular causes comprise less than 20% of torticollis cases in children. However, the frequency of osseous etiology was not evident [20,33]. Although the present study showed that 1.0 % of patients with abnormal head and neck posture had congenital osseous torticollis, the incidence of osseous torticollis with only head and neck posture abnormalities should be studied further.

This study has some limitations that should be acknowledged. First, this paper is a single-institution study. Further large-scale multicenter studies and analyses should be carried out to confirm our findings. Additionally, our study was conducted through a retrospective chart review. Among the children who visited our hospital with suspicion of congenital torticollis, our enrollment data were of the children who finished the congenital torticollis test protocol such as USG, cervical X-ray, and ophthalmologic examination within the period. Therefore, these data may not show the exact incidence. However, as mentioned earlier, there is lack of study presenting the importance of diagnosing osseous torticollis that mimics the most widespread diagnosis, CMT. Thus, our research has strength in that it aroused the awareness of the importance of diagnosing osseous torticollis, which could be mistaken for CMT in clinical practice.

## 5. Conclusions

In conclusion, congenital torticollis can be caused by a congenital vertebral anomaly, and the only symptom of osseous torticollis in children may be abnormal head and neck posture. It may be difficult to detect vertebral anomalies by physical examinations and X-ray findings, especially when the degree of invasion is only one level and only the head and neck postures are abnormal. Therefore, when ambiguous vertebral abnormalities are observed on cervical X-rays, clinicians should perform careful examinations and can benefit from additional examinations like 3D-CT of the cervical spine to avoid misdiagnosis.

## Figures and Tables

**Table 1:** General characteristics of subjects (*N* = 10).

Characteristic	Value
Age at the time of the first visit	1 month to 14 months
Age at the time of the diagnosis	
<1 year	9 (90.0)
≥1 year	1 (10.0)
Male:female	1 (20.0):4 (80.0)

Data are presented as range, number, or ratio (percentage).

**Table 2:** Types of anomaly of the cervical or thoracic vertebrae (*N* = 10).

Type of anomaly	Fusion only	7 (70.0)
	Multiple	3 (30.0)
Number of involved vertebra		
1 level		8 (80.0)
>1 level		2 (20.0)
Involved vertebra		
C2		5 (50.0)
C3		6 (60.0)
C4		2 (20.0)
C5		1 (10.0)
C6		2 (20.0)
C7		2 (20.0)
T1		4 (40.0)
T2		2 (20.0)

Data are presented as number (%).
